# Fracture Load of Polyaryletherketone for 4-Unit Posterior Fixed Dental Prostheses: An In Vitro Study

**DOI:** 10.3390/jfb16120448

**Published:** 2025-11-29

**Authors:** Dalea M. Bukhary, Hasan Y. Asiri, Ruwaida Z. Alshali, Walaa A. Babaeer, Thamer Y. Marghalani, Ghadeer I. Basunbul, Osama A. Qutub

**Affiliations:** Oral and Maxillofacial Prosthodontics Department, Faculty of Dentistry, King Abdulaziz University, P.O. Box 80209, Jeddah 21589, Saudi Arabia; hasiri0091@stu.kau.edu.sa (H.Y.A.);

**Keywords:** PEEK, PEKK, zirconia, milling, CAD-CAM, fracture load, fixed partial dentures, fracture pattern, four-unit FDP

## Abstract

**Background/Objectives**: This study aimed to evaluate the mechanical properties, particularly the fracture load, modulus of elasticity, and fracture patterns, of four-unit posterior tooth-supported fixed dental prostheses (FDPs) fabricated from various computer-aided design/computer-aided manufacturing (CAD-CAM) materials. Understanding the mechanical behavior of these materials is crucial for optimizing prosthesis performance in high-load-bearing posterior regions. **Methods**: A total of 68 standardized FDP frameworks were fabricated, each consisting of two abutments (first premolar and second molar) and two pontics (second premolar and first molar). The specimens were divided into four groups (*n* = 17): polyetheretherketone (PEEK), polyetherketoneketone (PEKK), 3Y zirconia (control 1), and 4Y zirconia (control 2). All samples underwent three-point bending tests using a universal testing machine with a crosshead speed of 0.5 mm/min. Fracture patterns were assessed visually and documented. Fractured specimens were examined using scanning electron microscopy (SEM). Data were analyzed using the SPSS v20. Normality was assessed with the Shapiro–Wilk test. The fracture loads were compared using the Kruskal–Wallis test with Bonferroni correction, and the modulus of elasticity was analyzed via a one-way ANOVA with Dunnett’s T3 post hoc test. A significance level of α = 0.05 was applied. **Results**: Significant differences were observed among the groups. The 3Y zirconia demonstrated the highest fracture load (2275 ± 511.03 N), followed by the 4Y zirconia (1034.28 ± 221.55 N), PEEK (883.21 ± 172.24 N), and PEKK (402.01 ± 127.98 N). PEEK showed ductile fracture behavior, while PEKK exhibited brittle failure. Both zirconia groups demonstrated brittle fracture patterns. **Conclusions**: PEEK and 4Y zirconia presented comparable fracture loads, but with differing fracture behaviors—ductile in PEEK and brittle in 4Y zirconia. The 3Y zirconia offered the highest fracture load, but with limited flexibility. PEKK showed the lowest mechanical performance. These findings highlight the importance of material selection for FDPs in posterior load-bearing areas, considering both fracture load and failure mode.

## 1. Introduction

The replacement of missing teeth is a central goal in restorative dentistry, often achieved using either fixed dental prostheses (FDPs) or removable partial dentures (RPDs) [1,2]. FDP, in particular, is widely used to restore dental function and aesthetics, typically spanning three to four units and supported either by natural abutment teeth or dental implants. Their success depends on multiple factors, including proper case selection, abutment condition, prosthesis design, and, crucially, the material used in their fabrication [2]. Traditionally, dental materials such as gold, metal alloys, and ceramics have been used in FDP fabrication. However, recent advances in material science and digital dentistry have introduced high-performance polymers such as polyetheretherketone (PEEK) and polyetherketoneketone (PEKK) into clinical practice. PEEK, a member of the polyaryletherketone (PAEK) family, has attracted attention due to its favorable properties, including a modulus of elasticity close to that of cortical bone, radiolucency, high wear resistance, and excellent machinability [3].

PEEK restorations can be fabricated using various methods, each affecting the material’s properties and clinical performance. Traditional subtractive manufacturing involves CAD/CAM milling from industrially pressed PEEK blanks, ensuring consistent mechanical characteristics. Pressing techniques use thermoplastic PEEK granules molded under high heat and pressure. Recently, additive manufacturing methods like 3D printing have emerged, allowing for customized designs with complex geometries and minimal material waste [4]. These characteristics make PEEK particularly attractive for use in the posterior region, where strength and stress distribution are critical [5,6,7,8]. Fixed dental prostheses are considered long-span if they are replacing more than one tooth. Long-span FDPs are subject to increased flexural forces and stress on the abutments, making material choice even more critical for long-term clinical success. While several studies have demonstrated the promising mechanical behavior of PEEK in three-unit FDPs, evidence on its suitability for four-unit applications remains limited [9,10,11]. Previous studies have reported that CAD/CAM-milled PEEK FDPs display higher fracture loads than those fabricated by other techniques, with deformation behaviors ranging from ductile to brittle depending on processing methods [12]. For instance, Rodríguez et al. reported in an in vitro study that PEEK outperformed zirconia in fracture resistance in their in vitro comparisons [10]. Demirci et al. compared several CAD/CAM materials—including PEEK, PEKK, fiber-reinforced composites (FRCs), and zirconia—and found that while zirconia exhibited the highest fracture load, PEEK and PEKK showed ductile failure modes, which may reduce the occurrence of catastrophic failures in clinical use [9].

Both PEKK and PEEK are the two most well-known of the polyaryletherketone (PAEK) family. Although structurally similar to PEEK, PEKK may behave differently due to its higher glass transition temperature and altered crystallinity. Compared to PEEK (both pure and glass-reinforced), PEKK demonstrated superior mechanical performance, particularly in flexural, tensile, and compressive strength [13,14,15]. PEKK was included in this study as a comparative material due to its similarity to PEEK and its potential as an alternative high-performance polymer for FDP frameworks. Despite these insights, the current literature lacks a comprehensive evaluation of four-unit posterior FDPs made from PEEK, especially under conditions simulating oral loading [16,17].

This comparative gap presents a critical barrier to informed material selection in demanding posterior prosthetic cases. To address this, the present study evaluated and compared the mechanical properties—specifically the fracture load and fracture patterns—of four-unit posterior FDP frameworks fabricated from PEEK, against PEKK and zirconia. Therefore, the null hypothesis of the study is that there are no significant differences in the fracture load, flexural modulus, and pattern between four-unit FDP frameworks fabricated from three dental materials: PEEK, PEKK, and zirconia. By simulating clinical loading conditions through in vitro testing, the study aimed to generate clinically relevant data that can guide prosthodontic decision-making for four-unit restorations. The findings are expected to provide new insights into the structural performance of emerging polymer-based materials in comparison to established ceramics, thereby contributing to the development of safer and more durable prosthetic solutions in teeth rehabilitation.

## 2. Materials and Methods

### 2.1. Study Design

This in vitro experimental study evaluated the mechanical performance of four-unit posterior fixed dental prostheses (FDPs) fabricated from four CAD/CAM milled materials: PEEK, PEKK, 3Y zirconia, and 4Y zirconia. Fracture load was measured using a standardized static loading (three-point bending) test, followed by a post-fracture microscopic analysis to characterize failure patterns.

### 2.2. Grouping and Sample Size Calculation

A pilot test was conducted using 12 FDP frameworks (*n* = 4 per group: PEEK, PEKK, and zirconia) to estimate effect size. The specimens were divided into the following: Group 1—PEEK, Group 2—PEKK, and Group 3 (control)—zirconia. Based on this, the sample size was calculated using the G*Power version 3.1.9.6, with an effect size of 0.4, significance level α = 0.05, and power = 0.80, determining that 17 specimens per group were required [18].

### 2.3. Virtual Die Model and Framework Design

**Figure 1:**
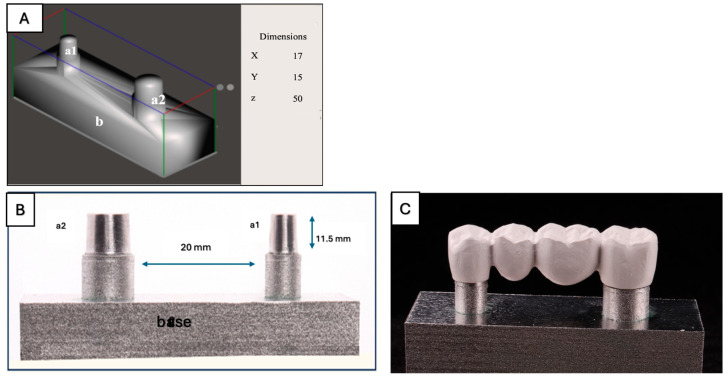
Virtual die model design and milling. (**A**) The design of the model was made in Exocad software. Dimensions of the die model: Length (z: 50), width (x: 17), and height (y: 15) in mm. Dimensions of the abutments (P: premolar, M: molar); height (11 mm) and distance between the two abutments (20 mm). (a1) first premolar abutment, (a2) second molar abutment, (b) die base. (**B**) Milled cobalt–chrome (type IV) die model, showing a 2-abutment die (11.5 mm height, 6° taper, 1 mm shoulder). (a1) prepared first premolar abutment, (a2) prepared second molar abutment; (b) pontic space for two teeth (20 mm), and a base measuring (50 mm × 15 mm × 17 mm). (**C**) 4-unit FDP framework placed on the dice model.

The virtual die model for a posterior 4-unit FDP was designed using the Autodesk Meshmixer (version 3.5.474). The model simulated a mandibular posterior edentulous span replacing the second premolar and first molar. Two abutments—the first premolar and second molar—were digitally prepared with a 11.5 mm height, 6° taper, 1 mm shoulder finish line, and a 20 mm interabutment distance (pontic space). The virtual model base measured (50 mm × 15 mm × 17 mm), ensuring compatibility with the universal testing machine. Then the FPD framework was designed in a dental CAD/CAM design software, Exocad (version 3.1), with a uniform 1.5 mm thickness, a rounded connector with areas of 19.9 mm^2^ for the central connector, 23.6 mm^2^ for the distal connector, and 16.8 mm^2^ for the mesial connector. The connector dimensions were adapted from established zirconia connector guidelines to accommodate polymer frameworks, ensuring adequate load distribution [19]. Each FDP was positioned on the cobalt–chrome die model without cementation to ensure consistent seating. A 70 μm internal space simulated a clinical cement space (Figure 1 and Table 1) [20].

#### Virtual Die Model and Framework Milling and Sample Preparation

All frameworks and die designs were exported as standard tessellation language (STL) files. The STL file of the die model was milled using a 5-axis Coritec 350i unit (imes-icore GmbH, Spenge, Germany) from cobalt–chrome (type IV alloy) (Figure 1C). The STL files of the frameworks were milled using the same 5-axis milling machine (Coritec 350i, imes-icore GmbH, Spenge, Germany) from 4 tested dental materials: Milled PEEK, Milled PEKK, and pre-sintered 3Y/4Y zirconia (Dental Direkt GmbH, Spenge, Germany) (Table 2).

After milling, sprues were removed from the milled cobalt–chrome die model, all surfaces were inspected for defects under 10× magnification, and no polishing was performed to maintain surface consistency across samples. The zirconia frameworks were sintered at 1500 °C for 4 h (Lava Therm, 3M ESPE), steam-cleaned, and sandblasted with 150 µm Al_2_O_3_ at 2 bar pressure for 10 s. The sandblasting procedure was standardized using an AL_2_O_3_ airborne-particle-abrasion unit with a 2 mm nozzle diameter. The nozzle was fixed at a 10 mm distance from the specimen surface and oriented perpendicularly (90°). The same operator performed all procedures under constant parameters (150 ± 92 μm Al_2_O_3_ particles, 2 bar pressure, 10 s). The unit was calibrated before each session to maintain consistent output [21]. Neither PEEK nor PEKK samples were sandblasted after milling, to avoid surface damage. The milling unit was calibrated before each milling by a single laboratory technician.

All framework materials were finished according to the manufacturers’ recommended protocols to ensure consistent surface quality. PEEK and PEKK frameworks were adjusted using fine-grit diamond burs under low pressure, followed by sequential rubber polishers from coarse to fine, and a final high-gloss finish using diamond paste. Zirconia frameworks were finished under water cooling with fine diamonds and polished using the two-step Panther system (purple and white polishers), followed by diamond paste. Cobalt–chromium frameworks were smoothed with progressively finer silicon carbide polishers and then polished with a high-speed felt wheel and metal polishing paste; electropolishing was performed when needed to enhance surface smoothness. All polishing procedures were conducted under low pressure to prevent heat generation, and each framework was prepared until a uniform, high-gloss surface was achieved to standardize the finishing across all specimens.

### 2.4. Mechanical Testing

Each FDP in the 4 groups (*n* = 17) was positioned on the cobalt–chrome die model without cementation to ensure consistent seating. A 50 µm internal space simulated a clinical cement gap. All FDPs were tested under three-point bending conditions using a universal testing machine with a crosshead speed of 0.5 mm/min. An axial load was applied at the central groove of the molar pontic until fracture. The fracture was identified by a sudden drop in the load curve and visual inspection of the framework fracture. The load-to-fracture data were captured in Newtons (N) using the Bluehill^®^ Universal v4.47.

### 2.5. Fracture Pattern

The fracture pattern of the frameworks was evaluated using Burke’s classification system. Codes I–V were used to classify minimal to catastrophic structural damage [22]. It provides a useful framework for categorizing fracture types, which can help compare the mechanical performance and failure modes of different materials. The fracture patterns were categorized as follows: **Code I**, Minimal fracture or crack in the crown; **Code II**, Less than half of the crown lost; **Code III**, crown fracture through midline (half of the crown displaced or lost); **Code IV**, more than half of the crown lost; and **Code V**, severe fracture of the crown and/or tooth.

### 2.6. Scanning Electron Microscopy (SEM)

Post-fracture surfaces were analyzed using SEM (AURA100, Uiwang-si, Gyeonggi-do, Republic of Korea) under the secondary electron mode at 10.0 kV, 9.1 mm WD, and spot size 1 mm.

### 2.7. Statistical Analysis

Data were analyzed using the IBM SPSS Statistics v20. A Shapiro–Wilk test was used to assess normality. Fracture load data were analyzed using Kruskal–Wallis tests followed by post hoc Bonferroni correction, while modulus of elasticity data were analyzed using a one-way ANOVA followed by Dunnett’s T3 test. All tests were performed at a significance level of α = 0.05. The mean and standard deviation values were initially calculated for all sample groups (Table 3). The normality of the data was assessed using the Shapiro–Wilk test. For fracture load, the *p*-values were PEKK < 0.001, PEEK = 0.019, 4Y zirconia = 0.375, and 3Y zirconia = 0.006, indicating that most of the data were not normally distributed (*p* < 0.05). For the modulus of elasticity, the data were normally distributed, as the Shapiro–Wilk test showed *p*-values ≥ 0.105. The Kruskal–Wallis test was conducted to assess fracture load, while modulus of elasticity data were analyzed using a one-way ANOVA and Dunnett’s T3 post hoc test. Table 3 summarizes the data for fracture load and flexural modulus.

## 3. Results

### 3.1. Fracture Load Testing

**Figure 2:**
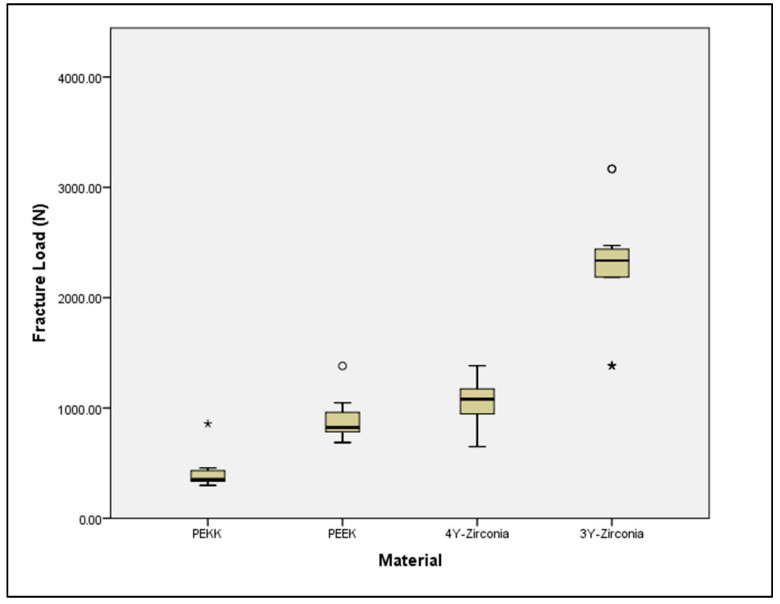
Box plot showing the distribution of fracture load values for each tested material. For each group, the box represents the interquartile range, the horizontal line inside the box shows the median, the whiskers indicate the data range, and the circles represent outliers. PEKK = polyetherketoneketone; PEEK = polyetheretherketone; 4Y zirconia = zirconia with 4 mol% yttria; and 3Y zirconia = zirconia with 3 mol% yttria. Asterisk (*) indicates outliers.

The results of the Kruskal–Wallis test revealed a statistically significant difference in fracture loads among the tested materials (*p* < 0.001). Pairwise comparisons showed that PEKK (mean fracture load = 402.01 N) exhibited significantly lower fracture loads compared to PEEK (883.21 N, *p* = 0.016), 4Y zirconia (1034.29 N, *p* < 0.001), and 3Y zirconia (2275.33 N, *p* < 0.001). PEEK also had significantly lower fracture loads compared to 3Y zirconia (*p* < 0.001). In contrast, the difference between PEEK and 4Y zirconia was not statistically significant (*p* = 0.242). Additionally, 4Y zirconia had significantly lower fracture loads than 3Y zirconia (*p* = 0.008) (Figure 2). Table 3 and Table 4 summarize the data for fracture load and modulus of elasticity.

**Figure 3:**
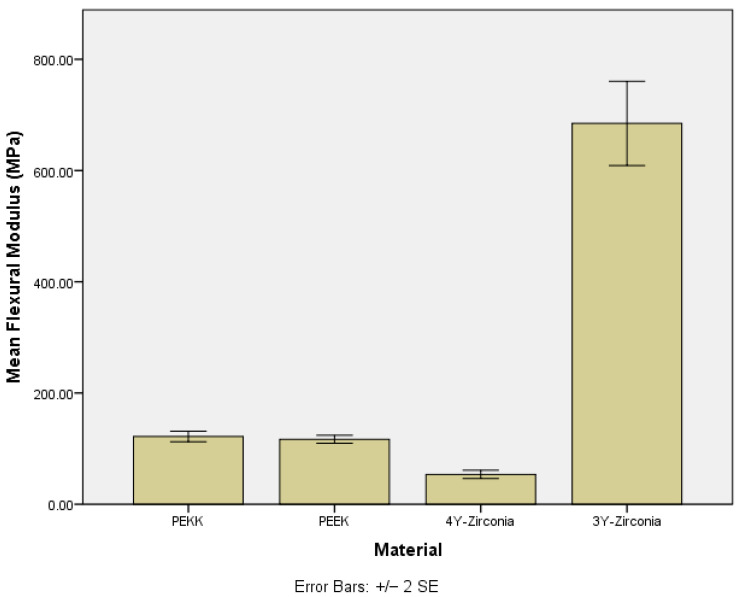
Flexural modulus among the tested materials. PEKK: polyetherketoneketone, PEEK: polyetheretherketone, 4Y zirconia: 4 mol% yttria-stabilized zirconia, and 3Y zirconia: 3 mol% yttria-stabilized zirconia. Error bars represent ±2 standard errors of the mean (±2 SE), calculated as the SD/$\sqrt{N}$ for each group.

The one-way ANOVA showed a statistically significant difference in the modulus of elasticity among the materials tested (F = 234.991, *p* < 0.001). The post hoc analysis using Dunnett’s T3 test revealed that 3Y zirconia had a significantly higher modulus of elasticity (mean = 684.73 MPa) compared to all other materials: PEKK (121.87 MPa, *p* < 0.001), PEEK (116.79 MPa, *p* < 0.001), and 4Y zirconia (53.70 MPa, *p* < 0.001). Additionally, PEKK and PEEK did not differ significantly from each other (*p* = 0.947), while both had significantly higher values than 4Y zirconia (PEKK vs. 4Y zirconia: *p* < 0.001; PEEK vs. 4Y zirconia: *p* < 0.001) (Figure 3).

The results indicated significant differences in mechanical performance across the materials. PEEK exhibited the highest flexibility, with a maximum deformation of 1.123 mm and an equivalent von Mises stress of 314.72 MPa. PEKK demonstrated lower deformation (1.125 mm) but experienced higher brittleness under stress, reaching 239.73 MPa. Zirconia showed the highest stress resistance, with maximum stress reaching 5083.6 MPa, though with a limited deformation capacity of 2.925 mm, as shown in Table 4.

**Figure 4:**
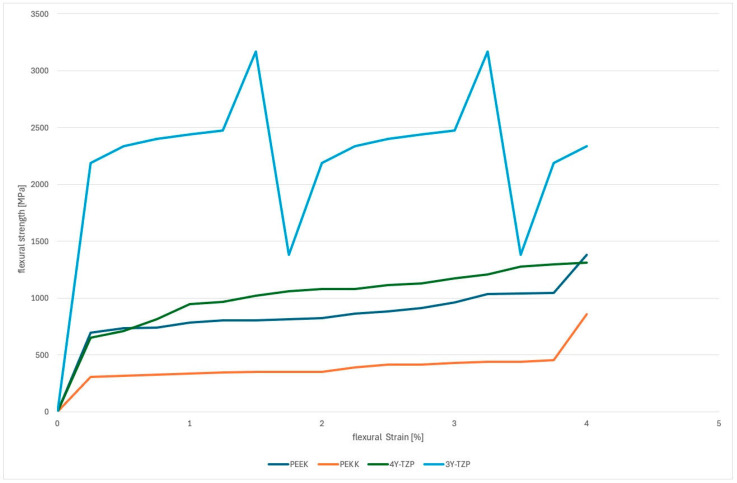
Representative flexural stress–strain curves of the tested materials. The diagram illustrates the relative deformation behavior of zirconia, PEKK, and PEEK under compressive loading.

Figure 4 presents a representative stress–strain curve for the tested materials, illustrating their overall deformation behavior under compressive loading. The flexural stress–strain curves demonstrated marked differences in mechanical response between the polymer-based materials (PEEK and PEKK) and the zirconia-based ceramics (4Y-TZP and 3Y-TZP). Both zirconia groups showed substantially higher compressive strength values across the full strain range, reflecting their inherently higher stiffness and load-bearing capability. The steep initial slope of the curves for 4Y-TZP, and especially 3Y-TZP, indicates a higher flexural modulus, which is consistent with the brittle, ceramic nature of yttria-stabilized zirconia. In contrast, the polymers (PEEK and PEKK) exhibited lower stress values at comparable strains, indicating lower stiffness, but with smoother and more progressive stress increase, which reflects a more ductile deformation mechanism. This behavior suggests that while zirconia can sustain high loads before catastrophic failure, PEEK and PEKK are capable of absorbing more deformation without sudden fracture. Overall, the curve profiles support that zirconia materials are mechanically superior in terms of compressive strength, whereas the polymer-based materials provide more elastic resilience.

### 3.2. Fracture Pattern

**Figure 5:**
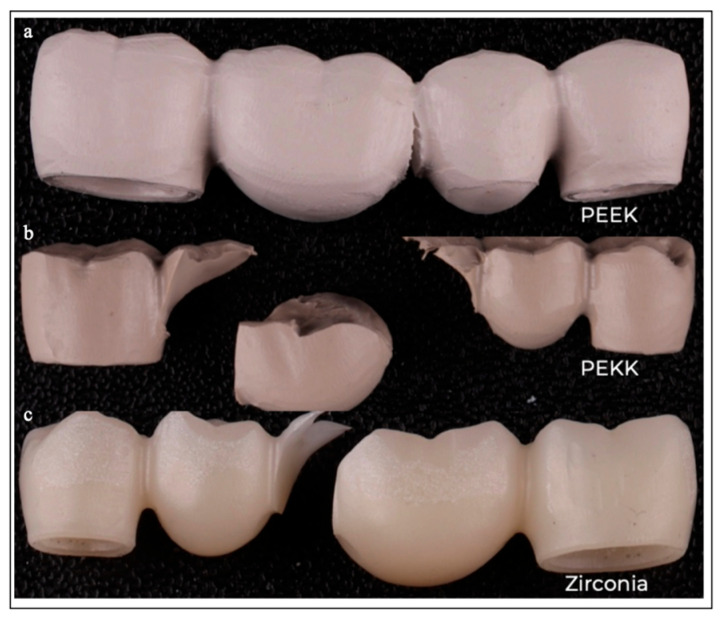
Framework fracture patterns according to Burke’s classification: (**a**) PEEK Code III (crown fracture through midline); (**b**) PEKK Code V (severe fracture of the crown and/or tooth); and (**c**) zirconia Code IV (more than half of the crown lost).

The failure patterns for each material were characterized as follows: zirconia was classified as Code IV, whereby more than half of the crown structure was lost, with typical brittle fragmentation. PEKK was categorized as Code V, which describes a severe fracture involving the crown and potentially extending into the tooth structure, with catastrophic structural collapse. PEEK was classified as Code III because the fracture was along the midline of the crown, with partial continuity preserved and ductile deformation observed (Figure 5).

### 3.3. Scanning Electron Microscopy (SEM)

**Figure 6:**
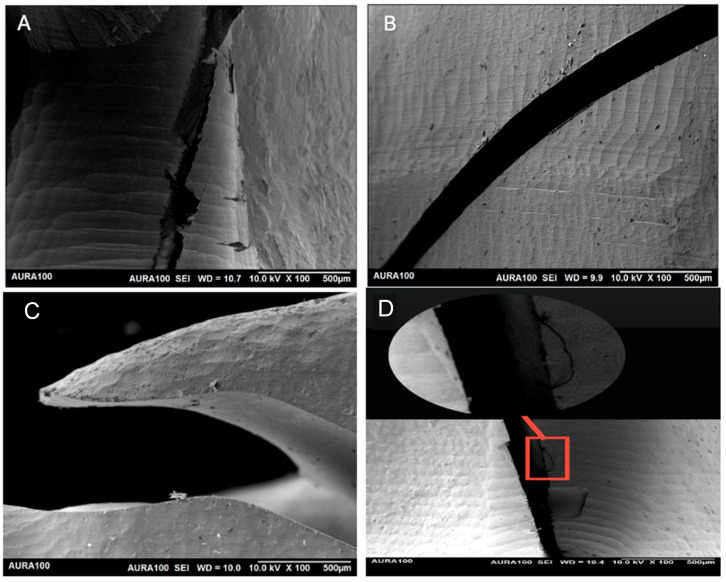
Scanning electron microscopy images showing the fracture lines of the tested materials: (**A**) PEEK; (**B**) PEKK; (**C**) 3Y Zirconia; and (**D**) 4Y Zirconia.

The SEM analysis revealed distinct microstructural fracture characteristics (Figure 6). PEEK exhibited ductile fracture characteristics, including evidence of tearing (Figure 6A). PEKK demonstrated a clean, straight fracture pattern, highlighting its brittle and fragile nature (Figure 6B). In addition, zirconia exhibited fracture features typical of a highly brittle ceramic. The SEM analysis revealed sharp fracture edges and a granular, river-pattern morphology without signs of plastic deformation, indicating catastrophic failure under stress. A granular morphology is characteristic of brittle ceramics and indicates unstable crack propagation [23,24,25].

These microscopic features support its potential clinical performance in scenarios requiring deformation tolerance without compromising on structural integrity.

## 4. Discussion

This in vitro study evaluated the mechanical behavior of four-unit posterior FDP frameworks fabricated from PEEK, PEKK, 3Y zirconia, and 4Y zirconia. All materials demonstrated distinct mechanical patterns, validating the rejection of the null hypothesis.

The materials exhibited substantial differences in fracture load, flexural modulus, and failure modes. Zirconia, particularly the 3Y type, demonstrated the highest fracture load (2275 ± 511 N), highlighting its superior mechanical strength.

In this study, the flexural modulus was calculated from the load–load-displacement curves and represents an apparent bending modulus of the FDP structure. Because the specimens were multi-unit frameworks subjected to a three-point bending-type loading configuration, the measured stiffness reflects the combined effect of material properties and the prosthesis’s geometry (span length, connector height, and cross-sectional thickness). This differs from the intrinsic Young’s modulus reported in the literature, which is obtained from standardized tensile tests on uniform specimens. As expected, the values obtained in this study are lower than the typical published moduli for bulk materials such as PEEK (3–4 GPa), PEKK (3–5 GPa), and zirconia ceramics (approximately 200–210 GPa). There have been reports on Young’s modulus for 4Y zirconia: 190–200 GPa, slightly lower than 3Y due to an increased cubic phase [26].

PEKK recorded the lowest fracture load (402.01 ± 127.98 N) and exhibited brittle failure patterns, indicating that it may not be suitable for long-span FDPs. PEEK displayed a moderate yet clinically acceptable fracture resistance (883.21 ± 172.24 N) and showed ductile failure behavior, indicating better energy absorption under stress. PEEK and PEKK are semi-crystalline thermoplastic polymers with inherent ductility and a relatively low elastic modulus compared with ceramic materials [27,28]. PEKK, although similar in composition, typically exhibits a higher degree of crystallinity and a more rigid backbone, which reduces deformation capacity and leads to lower fracture resistance [28]. The 3Y-TZP zirconia (3 mol% yttria) contains a high tetragonal phase content that enables transformation toughening, significantly increasing its strength and fracture resistance [29]. This explains why 3Y zirconia often exhibits the highest fracture load values among dental materials. In contrast, 4Y zirconia incorporates more cubic phase content, which improves translucency but decreases the transformation toughening mechanism and reduces flexural strength compared with 3Y zirconia [29,30]. This explains why 4Y zirconia performed better than the polymeric materials but was weaker than 3Y zirconia [27,28].

The variations in mechanical performance among studies can be attributed to multiple factors, including material source, prosthesis span, CAD/CAM protocols, and thermocycling procedures. For instance, Rodriguez et al. reported that, using steel die abutments, significantly higher fracture loads were observed for PEEK (3132.27 N) and zirconia (1859.95 N), likely due to differences in material brand and testing setups [8]. Longer pontic spans in our study (four-unit FPDs) contributed to reduced rigidity and increased bending, which likely decreased the fracture load compared to the shorter spans used in other studies [31].

Die model design played a critical role in load distribution. Our metal-based cobalt–chromium die model provided high rigidity and repeatability for mechanical testing, differing from other models that incorporated periodontal ligament simulation or less rigid base materials. Such differences in abutment design, span configuration, and test simulation can significantly influence fracture behavior and load capacity [8,16,22]. A study reported that increasing the elastic modulus of the die abutment may increase fracture load values [32].

In this study, the die material selected was cobalt–chromium because, as reported by Sadeq et al. (2021), die materials with a higher elastic modulus provide more stable and reproducible fracture load values by minimizing substrate deformation [33]. Using a highly rigid die reduces the risk of abutment flexure influencing fracture resistance measurements. Furthermore, compared with the commonly used stainless steel material, cobalt–chromium offers greater stiffness and hardness. Co–Cr alloys demonstrate a higher elastic modulus (210 GPa) than stainless steel, indicating superior rigidity and lower susceptibility to deformation under load [34,35].

In our study, the PEEK FDP framework was not cemented on the die model abutments during fracture load testing. The die model was made of a cobalt–chromium base metal alloy, which may affect the results. It was used to provide controlled, reproducible conditions in the study, and it does not replicate clinical biomechanics. The cobalt–chromium die has high elastic modulus. A rigid die has no abutment movement, and the stresses are concentrated within the framework, which may lead to an increase in the measured fracture load. Unlike the use of low-modulus dies such as resin or the dentin analog, which may have micromovements, leading to reduced fracture load values, The FDPs were designed with a 50 μm internal space to simulate a clinical cement gap but were not cemented, allowing passive and consistent seating across all specimens. This approach aimed to eliminate variability from cementation and focus solely on the intrinsic fracture resistance of the materials. While cementation may play a significant role in stress distribution clinically, its exclusion in this study allowed for a more controlled comparison. Frameworks were intentionally not cemented to avoid variability from cement thickness and seating pressure and ensure consistent baseline seating, and to evaluate only intrinsic material behavior. This constitutes a controlled comparison, but does not reflect clinical conditions where cementation of the FDP may improve load distribution.

Scanning electron microscopy (SEM) analyses further confirmed the distinct fracture patterns. PEKK showed clean, brittle fracture lines, which may necessitate design modifications or reinforcement to mitigate the risk of sudden fracture. This has been shown in previous studies [36,37].

Overall, the findings indicate that both PEEK and zirconia are promising materials for FPD frameworks, offering different mechanical benefits depending on clinical priorities. PEEK’s flexibility and fracture behavior suggest its potential as an alternative to 4Y-zirconia in certain scenarios. However, PEKK’s low fracture load limits its application in long-span restorations unless additional support measures are employed. Further in vitro fatigue testing and subsequent clinical studies are recommended to validate these findings under functional conditions.

This study has several limitations. Being in vitro, all tests were performed in controlled static lab settings, which do not replicate the complex oral and periodontal ligament environment. Factors like aging and varying occlusal forces were not fully simulated. The use of a three-point bending test, while useful for standardized comparison, does not reflect the full range of stresses seen in clinical conditions. Additionally, the study only assessed immediate fracture load and patterns, without examining long-term fatigue under cyclic loading. Although PEEK is susceptible to hydrothermal softening under extreme conditions, recent in vitro evidence indicates that its mechanical properties remain stable under intra-oral-like aging. For example, Priya et al. (2023) found that milled PEEK retained a flexural strength of ~3.8 GPa after extended thermocycling and aging [38]. Additionally, PEEK exhibits very low water absorption (~0.5%) and high chemical stability (Wang et al., 2022), which are favorable for long-term prosthetic frameworks [39]. Nonetheless, we recognize that full long-term clinical validation in FDP applications remains incomplete [38,39].

This limits insights into the materials’ durability over time. Future research should explore the fatigue behaviors of PEEK, PEKK, and zirconia under cyclic loading and evaluate how different connector designs respond to these forces to improve strength and longevity in multi-unit prostheses.

## 5. Conclusions

Within the limitations of this in vitro study, we observed the following:

1. 3Y zirconia demonstrated the highest fracture resistance but failed in a brittle and catastrophic manner, confirming its suitability for high-stress posterior applications where rigidity is prioritized.
2. 4Y zirconia presented a more moderate fracture resistance than 3Y zirconia, making it a reasonable compromise between strength and esthetics, but was mechanically inferior.
3. PEKK exhibited the lowest fracture resistance and brittle fracture behavior in FDP form, indicating that design modifications (larger connectors and reinforcement) are necessary for long-span applications.
4. Material selection for long-span posterior FDPs should balance fracture load, failure mode, and clinical priorities. Polymer-based frameworks may be advantageous where ductility and reduced catastrophic failure are desired.

## Figures and Tables

**Table 1:** Detailed dimensions of the connector design.

Connector	Height (mm)	Width (mm)	Area (mm^2^)
Mesial	4.728	4.211	19.909
Central	5.632	4.196	23.631
Distal	4.460	3.768	16.805

**Table 2:** Properties of the materials used in the study.

Materials	Manufacturers	Composition	Flexural Strength [MPa] According to Manufacturer	Material Specifications
PEEK	Dental Direkt GmbH Industriezentrum 106–108, 32139 Spenge, Germany	Polyetheretherketone ≥ 99.99 [wt%], further additives < 0.01 [wt%]	≥155	Lot no. 01060020 registered trademark of Cendres+Métaux Holding SA, Biel/Bienne, Switzerland.
PEKK	Cendres+Métaux SA Rue de Boujean 122 CH-250, Biel/Bienne, Switzerland	Polyetherketoneketone titanium dioxide	200	Lot no. 01060020 registered trademark of Cendres+Métaux Holding SA, Biel/Bienne, Switzerland.
3Y Zirconia	Dental Direkt GmbH Industriezentrum 106–108, 32139 Spenge, Germany	Zr$O_{2}$ + Hf$O_{2}$ + $Y_{2}O_{3}$ ≥ 99.0 [weight %], $Y_{2}O_{3}$ < 8 [weight %], ${AL}_{2}O_{3}$ < 0.15 [weight %], other oxides < 1 [weight %]	1200 ± 150	Lot no. 02_20221212 Dental Direkt GmbH Industriezentrum 106–108, 32139 Spenge, Germany, Tel.: +49-5225-863190
4Y Zirconia	Dental Direkt GmbH Industriezentrum 106–108, 32139 Spenge, Germany	Zr$O_{2}$ + Hf$O_{2}$ + $Y_{2}O_{3}$ ≥ 99.0 [weight %], $Y_{2}O_{3}$ < 8 [weight %], ${AL}_{2}O_{3}$ < 0.15 [weight %], other oxides < 1 [weight %]	700	Lot no. 02_20221212 Dental Direkt GmbH Industriezentrum 106–108, 32139 Spenge, Germany, Tel.: +49-5225-863190

**Table 3:** Means, standard deviations (SDs), maximum and minimum fracture load values (N), and flexural modulus (Mpa) of tested materials.

Material	N	Fracture Load Mean (N) *	SD	MIN	MAX	Flexural Modulus **	SD	MIN	MAX
**PEEK**	17	883.21 ^a^	172.24	687.01	1380.83	116.79 ^a^	14.87	77.94	139.41
**PEKK**	17	402.01 ^a^	127.98	298.64	856.86	121.87 ^a^	19.66	94.78	160.31
**3Y** **Zirconia**	17	2403 ^a^	497.15	1383.02	3167.33	694.07 ^a^	106.11	508.11	890.50
**4Y** **Zirconia**	17	1034.28 ^b^	221.55	650.35	1383.02	512.02 ^a^	96.11	408.11	790.50

* Newton; ** Mega Pascal; ^a^ statistically significant results ≤ 0.05; and ^b^ not statistically significant results > 0.05.

**Table 4:** The tested materials’ mechanical performances.

Material	Maximum Deformation (mm)	Maximum Equivalent Stress (MPa)	Strain Energy (mJ)	Observation
**PEKK**	1.1252	239.73	0.09821	Slightly more brittle than PEEK.
**PEEK**	1.1231	314.72	0.18463	High flexibility, moderate stress.
**Zirconia**	2.9251	5083.6	2.9781	Exceptional stress resistance but brittle.

## Data Availability

The original contributions presented in the study are included in the article, further inquiries can be directed to the corresponding author.

## Abbreviations

The following abbreviations are used in this manuscript:

PAEK	polyaryletherketone
PEEK	polyetheretherketone
ZrO_2_	zirconia
